# Reduced O-GlcNAcylation of SNAP-23 promotes cisplatin resistance by inducing exosome secretion in ovarian cancer

**DOI:** 10.1038/s41420-021-00489-x

**Published:** 2021-05-18

**Authors:** Luomeng Qian, Xiaoshan Yang, Shaohui Li, Hang Zhao, Yunge Gao, Shuhui Zhao, Xiaohui Lv, Xiyuan Zhang, Lingxia Li, Lianghao Zhai, Fuxing Zhou, Biliang Chen

**Affiliations:** 1 Department of Gynecology and Obstetrics, Xijing Hospital, Fourth Military Medical University, Xi’an, Shaanxi 710032 China; 2 State Key Laboratory of Military Stomatology and National Clinical Research Center for Oral Diseases and Shaanxi Key Laboratory of Oral Diseases, Center for Tissue Engineering, School of Stomatology, Fourth Military Medical University, Xi’an, Shaanxi 710032 China; 3 Department of Burns and Cutaneous Surgery, Xijing Hospital, Fourth Military Medical University, Xi’an, Shaanxi 710032 China; 4 Department of Cardiology, Xijing Hospital, Fourth Military Medical University, Xi’an, Shaanxi 710032 China

**Keywords:** Ovarian cancer, Ovarian cancer

## Abstract

Exosomes have been associated with chemoresistance in various cancers, but such a role in ovarian cancer is not yet clear. Here, using in vitro cell-based and in vivo mouse model experiments, we show that downregulation of O-GlcNAcylation, a key post-translational protein modification, promotes exosome secretion. This increases exosome-mediated efflux of cisplatin from cancer cells resulting in chemoresistance. Mechanistically, our data indicate that downregulation of O-GlcNAclation transferase (OGT) reduces O-GlcNAclation of SNAP-23. Notably, O-GlcNAcylation of SNAP-23 is vital for regulating exosome release in ovarian cancer cells. Reduced O-GlcNAclation of SNAP-23 subsequently promotes the formation of soluble N-ethylmaleimide-sensitive factor attachment protein receptor (SNARE) complex consisting of SNAP-23, VAMP8, and Stx4 proteins. This enhances exosome release causing chemoresistance by increasing the efflux of intracellular cisplatin.

## Introduction

Ovarian cancer is the leading cancer in women. Aggressive surgical debulking of the tumor and chemotherapy are the widely used treatment strategies^1,2^. However, due to chemoresistance, the five-year survival rate of ovarian cancer is around 47% for all stages^3,4^. Notably, the progression of tumor chemoresistance is a complex process involving multigenes, proteins, and factors^5^. Recently, the exosome, an important carrier of contents between the cells, is gaining serious attention for its role in tumor chemoresistance^6–8^. Previously, studies showed that compared to the normal cells, tumor cells secrete an increasing number of exosomes that transport key regulators to mitigate cell damage against chemotherapy^9^. Also, the growing evidence suggests that exosomes facilitate the efflux of intracellular chemotherapeutic drugs further reducing the damage to tumor cells eliciting chemoresistance^10^. However, the underlying molecular regulation of exosome secretion in ovarian cancer cells remains unclear.

The release of intracellular vesicles requires anchoring and fusion with the cytomembrane, a process that is regulated by specific proteins on the vesicle and the cytoplasmic membranes^11,12^. For instance, the secretion of exosomes is regulated by the SNAREs family of proteins^13,14^. A SNARE complex is composed of v-SNAREs and t-SNAREs proteins anchored on the vesicle and cytoplasmic membrane, respectively^15^. The interaction between these proteins drives vesicles to the cell membrane and ultimately promotes fusion to complete the vesicle secretion process^12,16^. SNAP-23, one of the t-SNAREs proteins, regulated by phosphorylation, facilitates the release of tumor cell exosomes by promoting stable complex formation with other SNARE proteins^17,18^. However, its role in regulating the release of cellular exosomes in ovarian cancer is not known yet.

O-GlcNAcylation, one of the important protein post-translational modifications, is known to regulate several biological processes^19^. Interestingly, OGT, the only known transferase of O-GlcNAcylation, has been reported for abnormal expression in various tumors^20^. Previously, we showed that downregulation of OGT increased cisplatin tolerance in the ovarian cancer cells^21^. Moreover, we found that the development of cisplatin resistance was linked to the O-GlcNAcylation level of SNAP-29, a t-SNARE protein. However, whether SNAP-23 is modified by O-GlcNAcylation and affects the secretion of exosomes is not known. Therefore, in this study, we aimed to investigate the role of OGT-mediated O-GlcNAcylation of SNAP-23 affecting exosome secretion in cisplatin resistance.

## Results

### Downregulation of OGT promotes the secretion of exosome in ovarian cancer cells

**Fig. 1:**
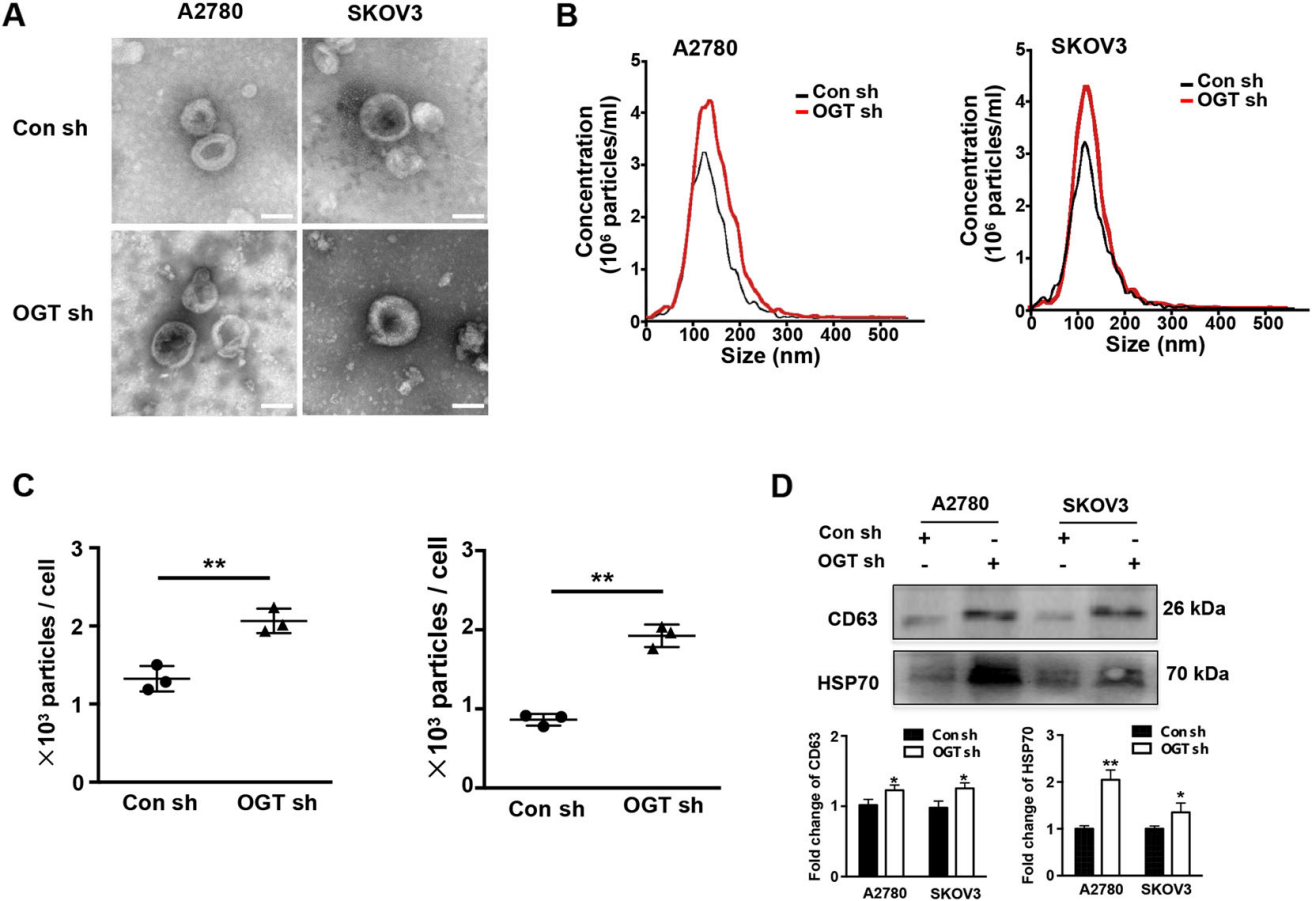
Downregulation of OGT promotes the secretion of exosome in ovarian cancer cells. **A** The morphology and size of exosomes in control and OGT-deficient cells were detected by TEM. Scale bars represent 100 nm. **B, C** The size distribution (B) and number (C) of two groups measured by NTA. **D** Western blotting analysis was used to test exosome markers CD63 and HSP70. The values are presented as mean ± SD (*n* = 3), which were three separate experiments performed in triplicate. **P < 0.01, *P < 0.05 (Student’s *t* test).

Current studies suggest that increased exosome secretion is related to tumor chemoresistance. Previously, we revealed that downregulation of OGT leads to cisplatin resistance in ovarian cancer. However, the relationship between OGT and exosome secretion is not well known. First, we established the lentiviral-based OGT knockdown in ovarian cancer cell lines A2780 and SKOV3 (Supplementary Figs). Then, the isolated exosomes from these were examined by TEM. We found that the exosomes from the OGT sh and Con sh groups exhibited similar morphology and size (Fig. 1A). Also, NTA indicated that the exosomes of both groups displayed similar size distribution (Fig. 1B). However, the number of exosomes in the OGT sh group was significantly higher than the control group (Fig. 1C). Furthermore, western blotting for exosome markers revealed that expression of CD63 and TSG101 was significantly higher in the OGT sh group (Fig. 1D). These results indicate that downregulation of OGT promoted exosome release in ovarian cancer cells.

**Fig. 2:**
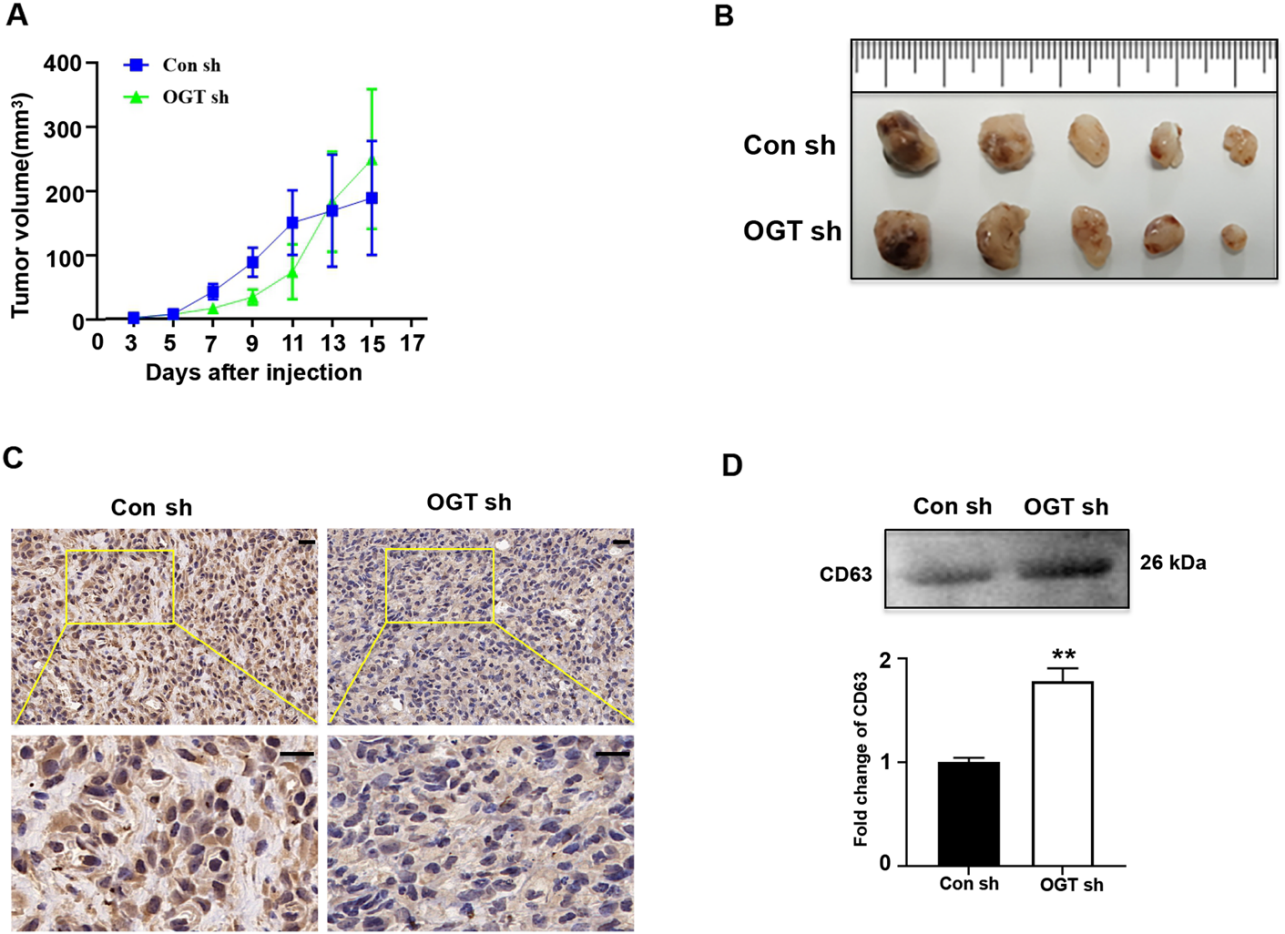
OGT deficiency leads to the increase of exosome secretion in vivo. **A** SKOV3 control and OGT-deficient cells were injected into the flanks of BALB/c nude mice. Tumor volume was measured every other day. Data are shown as mean ± SEM (*n* = 5 each group). **B** The volume and weight of subcutaneous xenograft tumors of SKOV3 cells isolated from BALB/c nude mice. **C** Expression of OGT in tumors tested by IHC. Scale bars represent 50 μm. **D** Western blotting analysis tested the expression of human CD63 protein expression level in nude blood from two groups. The values are presented as mean ± SD (*n* = 3), which were three separate experiments performed in triplicate. **P < 0.01 (Student’s *t* test).

To further examine the effect of downregulation of OGT on exosome secretion, we used a mice xenograft model. Notably, consistent with our previous results, downregulation of OGT did not affect the tumor formation time and size in the SKOV3 cells (Fig.  2A, B). Interestingly, after 15 days of tumor formation, immunohistochemistry evaluation revealed that OGT expression in the OGT sh group was significantly reduced (Fig. 2C). Next, the mice serum exosomes were examined for human CD63 protein expression levels in the two groups using human-specific CD63 antibody. Western blotting showed that the expression of human CD63 protein was significantly higher in the OGT sh group (Fig. 2D), indicating that downregulation of OGT promoted the release of exosomes from the ovarian cancer cells in vivo.

### OGT-mediated cisplatin resistance is linked to increased exosome secretion

**Fig. 3:**
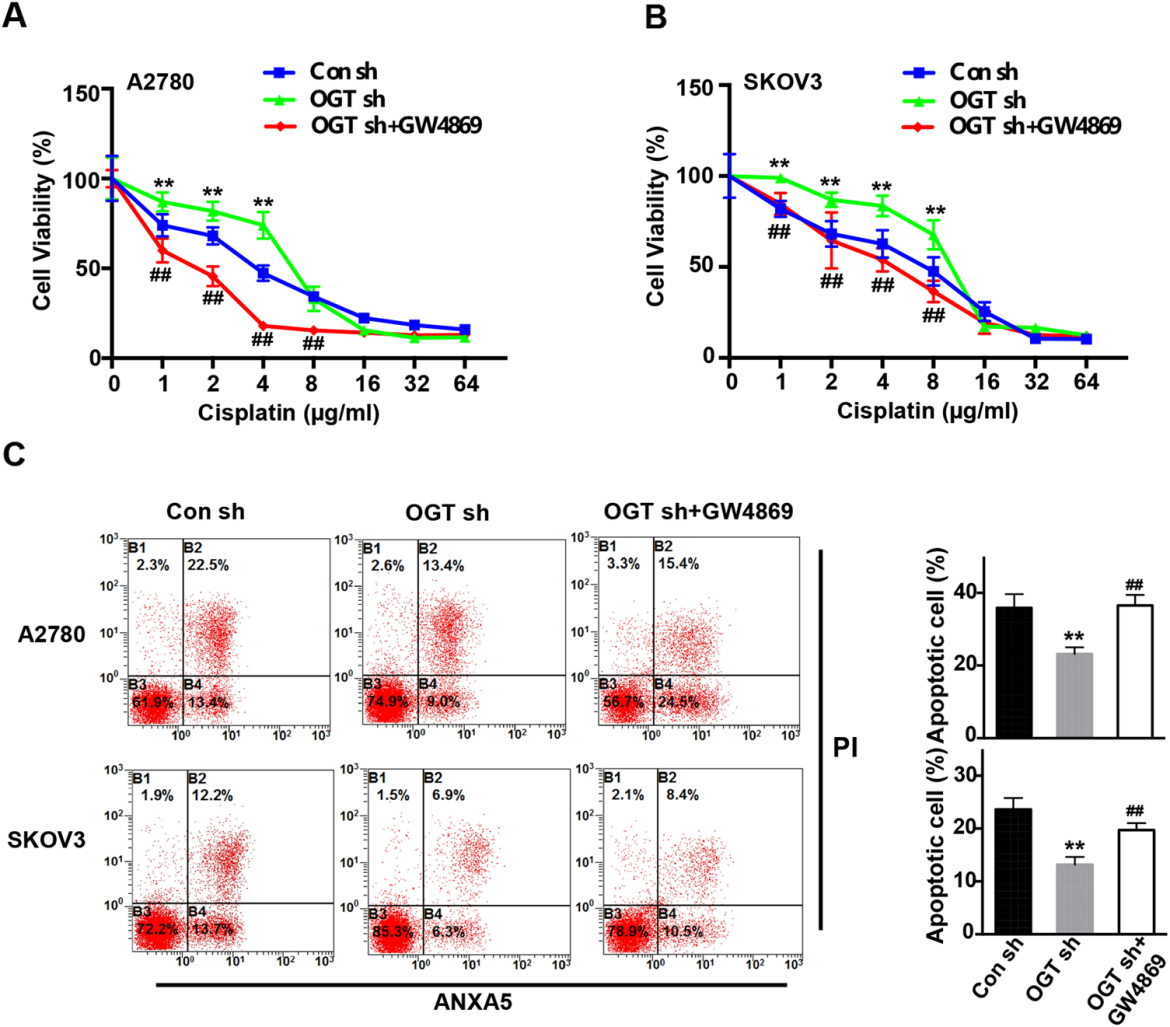
OGT-mediated cisplatin resistance is associated with increased exosome secretion. **A, B** Control, OGT-deficient cells and GW4869-treatment cells were treated with different concentrations of cisplatin for 24 h. The cell viability of A2780 (A) and SKOV3 (B) was measured by CCK-8 after cisplatin treatment. **C** Control, OGT-deficient cells, and GW4869-treatment cells were treated with cisplatin (5 µg/mL) for 24 h. Apoptotic cells were measured by ANXA5 and PI staining. The numbers shown are the sum of ANXA5-positive and double-positive cells. The values are presented as mean ± SD (*n* = 3), which were three separate experiments performed in triplicate. ^**^P < 0.01 vs Con sh, ^##^*P* < 0.01 vs OGT sh (Student’s *t* test).

To explore whether the increased exosome, caused by downregulation of OGT, are related to cisplatin resistance, we performed studies with GW4869, an inhibitor of exosome release. First, we treated ovarian cancer cells with cisplatin and found that downregulation of OGT significantly enhanced the viability of cisplatin-treated cancer cells (Fig. 3A, B). However, OGT-mediated cisplatin resistance was reversed by inhibiting exosome release (Fig. 3A, B). Furthermore, downregulation of OGT significantly reduced apoptosis in cisplatin-treated cells. This effect too was reversed by inhibition of exosome secretion (Fig. 3C). Overall, these results suggest that downregulation of OGT-mediated cisplatin resistance is linked to increased exosome release.

### Downregulation of OGT promotes cisplatin efflux by mediating exosome release

**Fig. 4:**
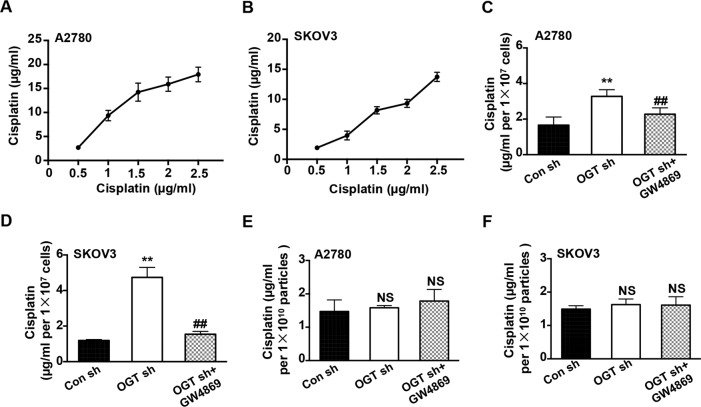
OGT deficiency promotes cisplatin efflux by mediating exosome release. ICP-MS was used to detect the concentration of cisplatin in A2780 and SKOV3 cells. **A, B** The presence of cisplatin in A2780 (A) and SKOV3 (B) exosomes with different concentrations of cisplatin. **C, D** The concentration of cisplatin in A2780 (C) and SKOV3 (D) exosomes of control, OGT-deficient cells, and GW4869-treatment cells. **E, F** The concentration of cisplatin in the same amounts of exosomes in each group of A2780 (E) and SKOV3 (F) cells. The values are presented as mean ± SD (*n* = 3), which were three separate experiments performed in triplicate. ^**^P < 0.01 vs Con sh, ^##^*P* < 0.01 vs OGT sh (Student’s *t* test).

Previously, several studies reported exosome-mediated chemotherapy resistance via increased efflux of chemotherapeutic drugs^10^. We estimated the presence of cisplatin in ovarian cancer exosomes by ICP-MS. The results revealed that the exosomes not only contained a considerable amount of cisplatin but also had increased concentration as the amount of cisplatin increased in the medium (Fig. 4A, B). It appears that downregulation of OGT significantly increased the cisplatin concentration in exosomes. Notably, inhibiting the release of exosomes reversed this phenomenon (Fig. 4C, D). Importantly, we found no significant difference in cisplatin concentration in the same amount of exosomes in each group (Fig. 4E, F). These results suggest that downregulation of OGT promotes cisplatin efflux, which is related to the increased exosome release.

### Downregulation of OGT increases exosome release by promoting secretion

**Fig. 5:**
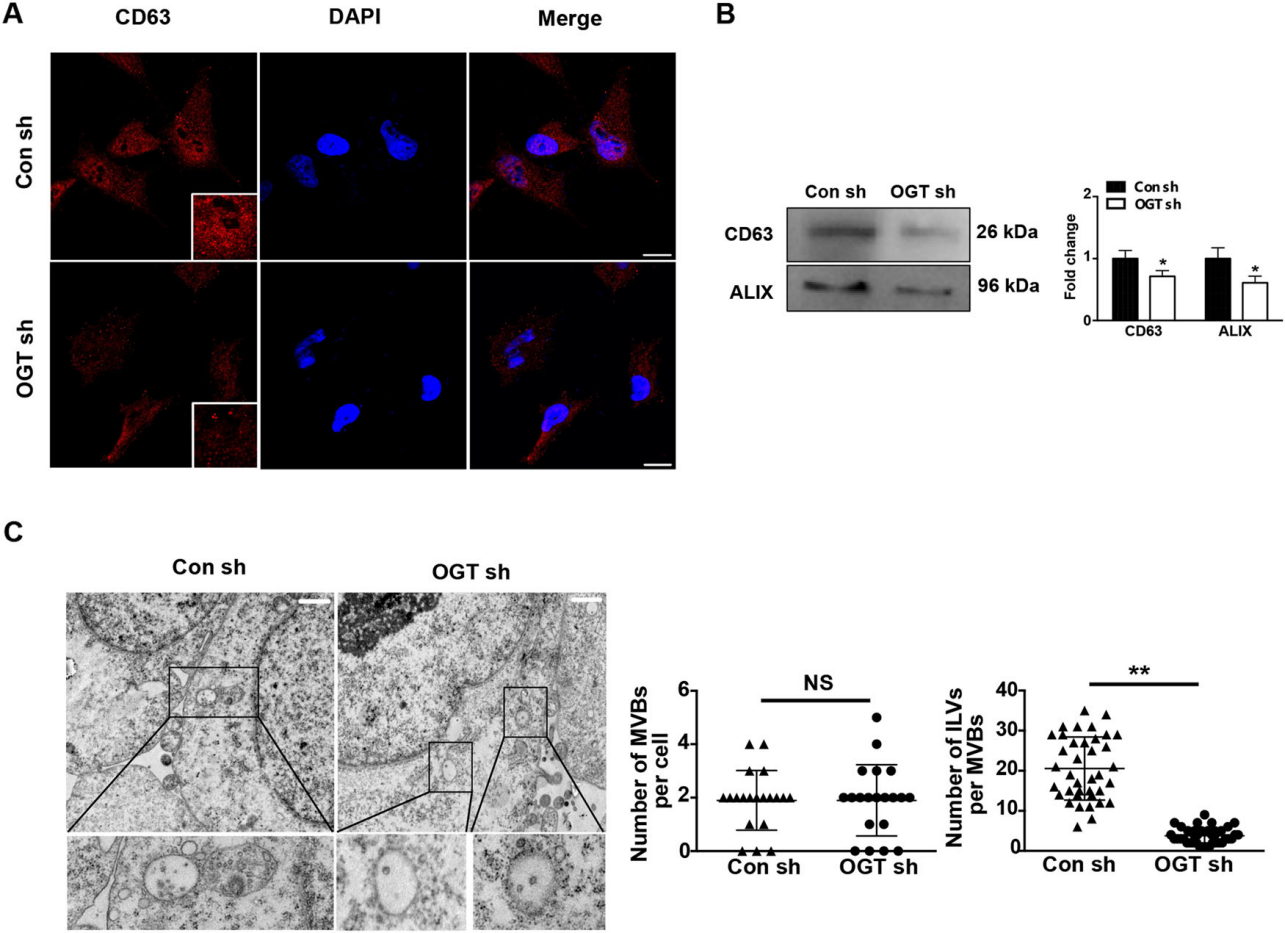
Down regulation of OGT increases the release level of exosomes by promoting the secretion process of exosomes. **A** The expression of CD63 in control and OGT-deficient cells was detected by immunofluorescence. Scale bars represent 10 μm **B** Western blotting analysis tested the expression of ILV markers CD63 and ALIX. **C** The number of MVBs and ILVs in MVBs of control and OGT-deficient cells was detected by transmission electron microscopy. Scale bars represent 10 μm. The values are presented as mean ± SD (*n* = 3), which were three separate experiments performed in triplicate. ^**^P < 0.01, ^*^P < 0.05 (Student’s *t* test).

Next, we examined whether the downregulation of OGT promotes exosome release by promoting exosome synthesis or secretion. For this, we examined CD63 expression in ovarian cancer cells by immunofluorescence and found that downregulation of OGT significantly reduced the fluorescence intensity of CD63 (Fig. 5A). Similar conclusions were obtained by western blotting. In these experiments, western blotting was performed to detect ILV (intraluminal vesicle) marker, ALIX. The results indicated that ALIX was significantly downregulated in the OGT sh group (Fig. 5B). Moreover, TEM showed that after OGT knockdown, the number of multivesicular bodies (MVBs) did not change significantly. However, the number of intraluminal vesicles (ILVs) in MVBs decreased significantly (Fig. 5C). These results suggest that downregulation of OGT promotes the exosome secretion.

### OGT-induced aberrant exosome secretion is linked to SNAP-23

**Fig. 6:**
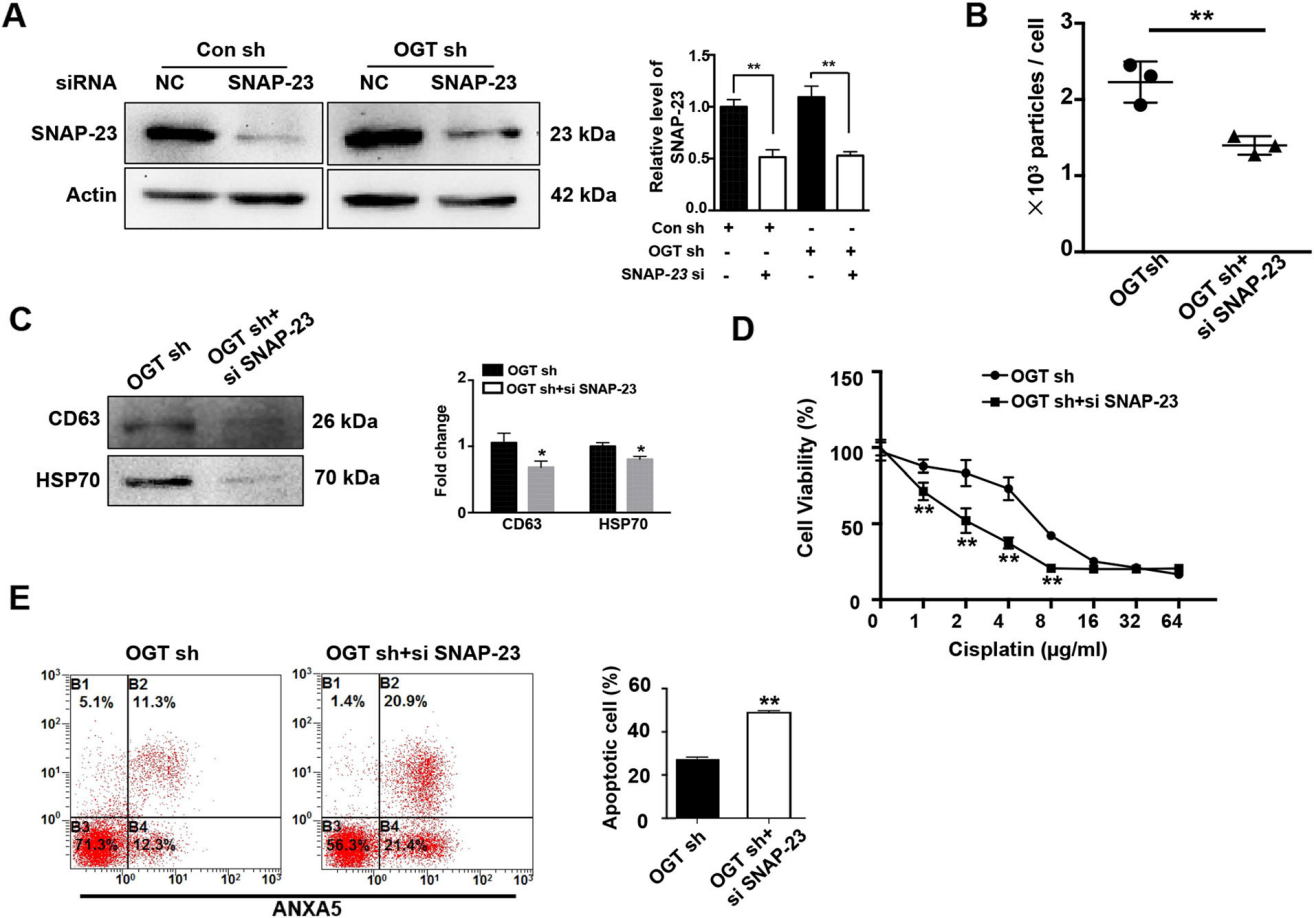
Aberrant exosome secretion induced by OGT deregulation is associated with SNAP23. **A** Control and OGT-deficient SKOV3 cells were transfected with NC siRNA or SNAP-23 siRNA and the expression levels of SNAP23 were detected by western blot analysis. **B** The release level of exosomes in OGT sh and OGT sh + si SNAP-23 group measured by NTA. **C** Western blot analysis tested the expression levels of CD63 and HSP70. **D** OGT sh and OGT sh + si SNAP-23 group cells were treated with different concentrations of cisplatin for 24 h. The cell viability was measured by CCK-8. **E** OGT sh and OGT sh + si SNAP-23 group cells were treated with cisplatin (5 µg/mL) for 24 h. Apoptotic cells were measured by ANXA5 and PI staining. The numbers shown are the sum of ANXA5-positive and double-positive cells. The values are presented as mean ± SD (*n* = 3), which were three separate experiments performed in triplicate. ^**^P < 0.01, ^*^P < 0.05 (Student’s *t* test).

Several studies suggest that SNAP-23 plays a critical regulatory role in exosome release. However, it is dubious whether SNAP-23 is also involved in OGT-mediated regulation of exosome release. To test this, we performed siRNA-mediated knock down of SNAP-23 in the SKOV3 cells (Fig. 6A). We observed that downregulation of SNAP-23 significantly reduced the release of exosomes in the OGT sh group (Fig. 6B). Moreover, western blotting revealed that SNAP-23 knockdown significantly reduced the expression of CD63 and HSP70 as compared to the control group (Fig. 6C). Besides, the downregulation of SNAP-23 reversed the OGT-knockdown-induced resistance of cisplatin (Fig. 6D, E). Overall, these results indicate that the OGT-mediated release of exosomes involves SNAP-23.

### O-GlcNAcylation of SNAP-23 regulates exosome secretion

**Fig. 7:**
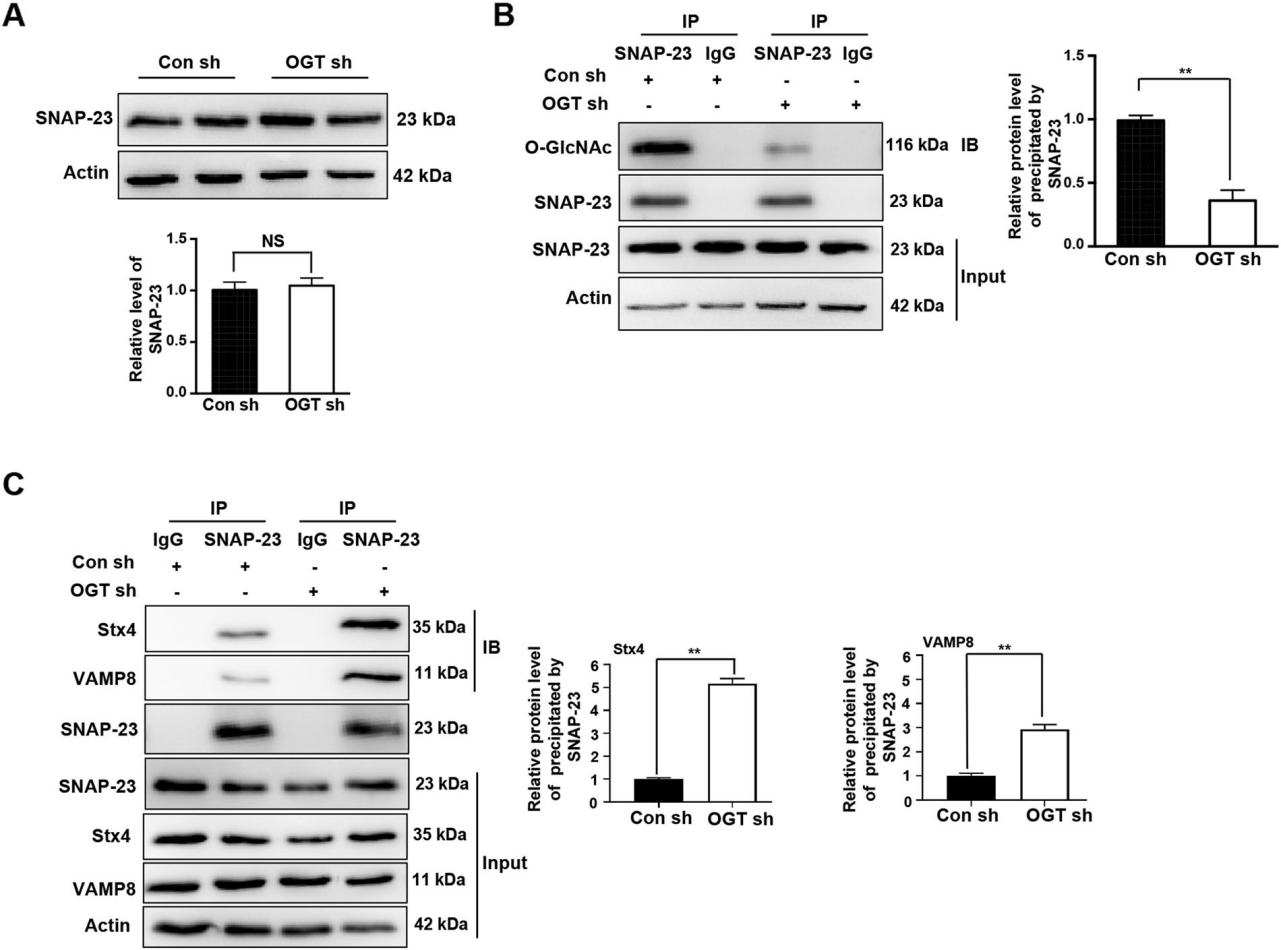
O-GlcNAcylation of SNAP-23 regulates the interaction of SNAP-23 with Stx4 and VAMP8 and promotes exosome secretion. **A** The expression of SNAP-23 in SKOV3 cells stably expressing control shRNA or OGT shRNA was tested by western blotting. **B** The control and OGT-deficient SKOV3 cell extracts were immunoprecipitated with anti-SNAP-23 and the resulting precipitants were immunoblotted against O-GlcNAc. **C** The control and OGT-deficient SKOV3 cells were immunoprecipitated with anti-SNAP-23 and the resulting precipitants were immunoblotted against Stx4 and VAMP8. Whole-cell lysates were tested for SNAP-23 and actin. The values are presented as mean ± SD (*n* = 3), which were three separate experiments performed in triplicate. ^**^P < 0.01 (Student’s *t* test).

To further explore the mechanism of OGT and SNAP-23-mediated regulation of exosome release, we examined SNAP-23 expression in the Con sh and OGT sh groups. Interestingly, we found no difference in SNAP-23 expression levels between the two groups (Fig. 7A). Previously, we showed that SNAP-29 was modified by O-GlcNAcylation^21^. COIP suggests that SNAP-23 could also be modified by O-GlcNAcylation (Fig. 7B). Meanwhile, we found that the O-GlcNAcylation level of SNAP-23 was significantly reduced in the OGT-knockdown group (Fig. 7B). Previously, studies showed that SNAP-23 regulates the exosome release along with Stx4 and VAMP8 (ref. ^23^). COIP revealed significantly increased coprecipitation of SNAP-23 with Stx4 and VAMP8 after the downregulation of OGT (Fig. 7C). The above results suggest that downregulation of OGT promotes the formation of the SNAP-23–Stx4–VAMP8 complex to increase exosome release.

## Discussion

The development of chemoresistance in ovarian cancer is a multifactorial regulation involving various mechanisms^24^. In this study, we show that ovarian cancer cells efflux cisplatin through exosomes, and the process is related to the intracellular O-GlcNAcylation level of regulatory proteins. The expression levels of OGT, a key O-GlcNAcylation transferase, directly affect the O-GlcNAc modification of intracellular proteins^25,26^. Previously, we showed that downregulation of OGT leads to cisplatin resistance^21^. Here, we revealed that downregulation of OGT promotes exosome release in ovarian cancer cells, which in turn increases the exosome-mediated efflux of intracellular cisplatin. This reduces the intracellular cisplatin concentration increasing cisplatin resistance in ovarian cancer.

Exosomes, bubble-like membranous structure vesicles with a diameter of 30–150 nm, are broadly present in most body fluids^27,28^. Exosomes are involved in the transport of a variety of proteins, chemicals, and microRNA (miRNA), mRNA, DNA, and other key signal molecules between the cells. This allows to regulate the physiological state of cells and has been closely linked to the occurrence of many diseases^29,30^. Studies show that tumor cells release more exosomes than normal cells and the contents of exosomes vary depending on the cell type^28^. Exosomes provide a suitable microenvironment for tumor cells and have been known to promote tumor proliferation^28,31^, chemoresistance^32^, metastasis^32^, and so on. Exosomes not only affect the tumor cells’ tolerance to chemotherapy by enabling the transport of regulatory substances, but also reduce cell damage by increasing the efflux of chemotherapeutic drugs. Dorayappan et al. showed that exosomes in the serum of drug-resistant patients were significantly higher than the drug-sensitive patients, and the exosome-mediated cisplatin efflux was higher after hypoxia in ovarian cancer cells^10^. In this study, we showed that downregulation of OGT did not change the morphology and size of exosomes, but significantly promoted their secretion. This led to increased efflux of cisplatin and reduced intracellular concentration of cisplatin causing cisplatin resistance in OGT-knockdown cells. This mechanism of cisplatin efflux seems to be more direct and faster. Understanding the mechanism of exosome-mediated efflux of cisplatin in cancer cells is clinically important to develop novel therapy for cisplatin-resistant ovarian cancer.

Exosome biogenesis is a multistep process involving MVB and ILV formation, transport of MVBs to the plasma membrane, and ultimately fusion with the plasma membrane^33^. Previous studies showed that the SNARE family of proteins regulates the final step of exosome secretion. MVBs fusion with the plasma membrane and release ILVs as exosomes^18,34^. Yang et al. showed that SNAP-23 induces MVB fusion and the exosome secretion, highlighting its essential role in exosome release^17^. Hu et al. showed that upregulated SNAP-23 promoted exosome secretion^35^. Along similar lines, in this work, we show that the effect of OGT downregulation on exosome secretion and cisplatin resistance was abolished upon treatment with si-SNAP-23 (siRNA). Previous studies showed that SNAP-23, VAMP8, and Stx4 composing the SNARE complex regulated membrane fusion and exosome secretion^23,36^. In the absence of any of these SNARE proteins, the complex formation would be prevented hindering exosome secretion^37^. Previously, we revealed that OGT and O-GlcNAc levels were linked to cisplatin resistance involving O-GlcNAcylated SNAP-29 (ref. ^21^). However, studies exploring the role of O-GlcNAc modification in regulating the interaction between SNAP-23, VAMP8, and Stx4 are not known. Our results showed that downregulation of OGT reduced O-GlcNAclation of SNAP-23. This promotes SNAP-23 interaction with Stx4 and VAMP8 facilitating SNARE complex formation to increase MVB and plasma membrane fusion and ultimately exosome secretion.

**Fig. 8:**
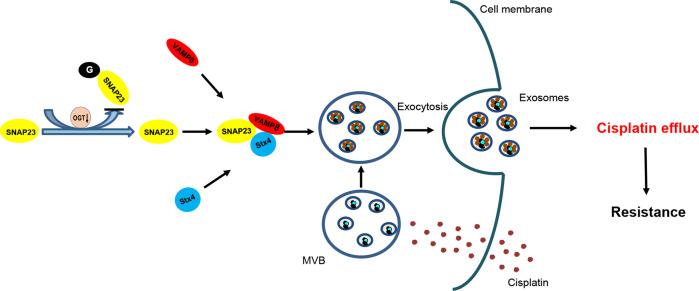
A schematic representation of a model. Downregulation of OGT promotes SNAP-23–Stx4–VAMP8 complex formation and exosome secretion, thus increasing the transport of intracellular cisplatin and reducing intracellular concentration of cisplatin, thereby improving the resistance of ovarian cancer cells to cisplatin.

In summary, our results demonstrate a vital role of OGT in regulating exosome secretion in ovarian cancer cells. Downregulation of OGT promotes exosome secretion that increases efflux of intracellular cisplatin. This leads to reduced intracellular concentration of cisplatin resulting in drug resistance in ovarian cancer. Moreover, downregulation of OGT reduces O-GlcNAclation of SNAP-23 and thereby promotes SNAP-23–Stx4–VAMP8 complex formation and exosome secretion. Our study highlights the function of exosomes in cisplatin resistance in ovarian cancer that can help manage drug-resistant ovarian cancer (Fig. 8).

## Materials and methods

### Cell lines and culture

Human ovarian cancer cells (A2780 and SKOV3) were commercially procured from the Shanghai Cell Collection (Shanghai, China). These were grown in RPMI 1640 (SH30809.01, Hyclone, Utah, USA) and McCoy 5 A medium (16600082, Gibco, New York, USA), 10% Fetal Bovine Serum (FBS, 11012-8611, Tianhang, Shandong, China) at 37 °C, and 5% CO_2_.

### Generation of stable cell lines and siRNA transfection

A2780 (80000 cells/well) and SKOV3 (100000 cells/well) cells were seeded into six-well plates and cultured with complete RPMI 1640 and McCoy 5 A medium for 24 h before lentiviral particle (encoding shRNA) treatment for 8 h. These were further incubated in a normal culture medium for 48 h. Finally, stable cell lines were established by selecting A2780 and SKOV3 cells using puromycin for 3–4 weeks. For knockdown studies, small-interfering RNAs (siRNAs) against SNAP-23 (sense: 5′-GGCUGACACCAACAGAGAUTT-3′, antisense: 3′-AUCUCUGUUGGUGUCAGCCTT-5′) were synthesized (GenePharm, Shanghai, China) and delivered into SKOV3 cells with Micropoly-transfecter (MT110, Micropoly, Jiangsu, China) according to the cell reagent protocol.

### GW4869 treatment of cell lines

GW4869 (HY19363, MCE, New Jersey, USA) was initially dissolved in DMSO (196055, MP Biomedicals, California, USA**)** to prepare a 10 mM stock solution. In cell culture condition, this was diluted to 20 µM (note: the final DMSO concentration was 0.005%).

### Isolation of exosomes

A2780 and SKOV3 cells were cultured in RPMI 1640 and McCoy 5 A medium without 10% FBS for 24 h. From these, cell culture supernatants were collected and centrifuged at 300 × g for 5 min. The collected supernatants, transferred into new tubes, were again centrifuged at 2000 × g for 20 min. Once more, to remove cells and cellular debris, these supernatants were transferred to new tubes and centrifuged at 10,000 × g for 30 min at 4 °C. Finally, the supernatants were transferred to clean centrifuge bottles (355618, Beckman Coulter, California, USA) and ultracentrifuged at 120,000 × g (TYPE 70 Ti, Beckman, California, USA) for 70 min at 4 °C. The obtained exosome pellets were resuspended in PBS and ultracentrifuged for another 70 min at 120,000 × g and 4 °C. The final product pellets were resuspended in 70 μl of PBS for further analysis.

### Nanosight nanoparticle tracking analysis (NTA)

The size and concentration of the exosome samples were estimated using the Zeta View instrument (Pmx110, Particle Metrix, Germany). For this, first, the exosome pellets were well resuspended and diluted in PBS within the recommended concentration range of 1 × 10^8^ particles/mL. Then, the samples were loaded into the Zeta View instrument for analysis at 405 nm. In total 30 pictures per second were obtained for 1 min. Subsequently, the resultant videos were analyzed with the NTA ZetaView 8.02.28 software to identify and track the center of each particle under Brownian motion. The average particle-traveled distance was measured on a frame-by-frame basis.

### Transmission electron microscopy (TEM)

The extracted exosomes were dissolved in 50–100-µl 2% paraformaldehyde solution. From this, 5–10-µl exosome solution was added to the Formvar-carbon sample copper net. A 100-µl PBS drop was set on the parafilm and the copper mesh (Formvar membrane facing down) was cleaned using the PBS droplet. Next, the copper mesh was put on a 50-µl 1% glutaraldehyde droplet for 5 min and then on 100-µl ddH2O for 2 minutes. Washing with water was performed eight times. After this, the copper mesh was put on a 50-µl uranyl oxalate drop (pH 7.0) for 5 minutes, then on a 50-µl methylcellulose drop for 10 minutes, performed on ice. Finally, the copper mesh was set on the stainless-steel ring on the top of the sample table and any excess of liquid was absorbed with filter paper, followed by air-drying for 5–10 min. Last, the electron microscope pictures were collected at 80 kV.

### Inductively coupled plasma mass spectrometer (ICP-MS)

The extracted exosomes were diluted with 2% dilute nitric acid and subjected to ICP-MS analysis (NexIONTM 350D, PerkinElmer, Massachusetts, USA). The instrument parameters were set to RF Power 1075 W, nebulizer gas flow 0.875 L/min, auxiliary gas flow 1.45 L/min, plasma gas flow 16.0 L/min, sample uptake rate ~0.8 ml/min, dwell time/AMU 100 ms, and integration time 10 s, in cell mode standard.

### Western blotting

A2780 and SKOV3 cells were lysed in protease inhibitor (AR1178, Boster, Wuhan, China) supplemented with RIPA buffer (P0013B, Beyotime, Jiangsu, China). The western blotting of the samples was performed as described previously^22^. Briefly, the proteins were separated by SDA-PAGE and transferred to PVDF membranes. These were overnight-incubated with primary antibodies at the appropriate concentration and 4 °C. The membranes were washed with PBST (phosphate-buffered saline Tween-20). Incubation with horseradish peroxidase-conjugated secondary antibodies was performed for 1 h at room temperature (RT). The protein bands were visualized using the ECLTM Western Blotting Detection Reagent (Bio-Rad, California, USA). The antibodies used in this study were as follows: OGT (1:1000, ab96718, Abcam, USA), O-GlcNAcylation (1:1000, ab2739, Abcam, USA), SNAP-23 (1:500, ab4114, Abcam, USA), ALIX (1:1000, ab27345, Abcam, USA), and Actin (1:1 000, bs-0061, Bioss, Beijing, China).

### Coimmunoprecipitation (CoIP)

The samples (each having 2 × 10^7^ cells) were lysed on ice by treating with NP40 lysis buffer (AR0107, Boster, China) for 20 min. The extracts were centrifuged at 12,000 × g for 20 min at 4 °C. Protein A/G PLUS-Agarose (SC-2003, Santa Cruz Biotechnology, China) was washed thrice with RIPA buffer. The supernatants were incubated overnight with primary antibodies at 4 °C. Then, the prepared antibody–agarose complex was added to 1 ml of whole-cell lysate, and the incubation was performed for 4 h at 4 °C. This was followed by three-time washing with NP40 lysis buffer. Finally, using the 2X SDS gel loading buffer, the protein samples were denatured by boiling for 5 min and subjected to immunoblotting.

### Immunocytochemistry

SKOV3 cells were grown in confocal dishes at 80% confluency and fixed with 4% paraformaldehyde. Then, these were permeabilized with 0.2% Triton X-100 for 15 min. The cells were blocked with serum for 1 h at RT and subsequently incubated overnight with anti-CD63 (1:100, 25682-1-AP, Proteintech, USA) at 4 °C. After washing thrice with PBS, the cells were incubated with the secondary antibodies for 1 h and DAPI for 30 min at RT. Last, the samples were washed thrice in PBS and examined under a fluorescence microscope (Olympus BX51TF, Japan).

### Cell viability assays

A2780 (5000 cells/well) and SKOV3 (8000 cells/well) cells were added to 96-well plates. These were treated with cisplatin (HY-17394, MCE, New Jersey, USA) or GW4869 at a concentration gradient (6 replicates for each concentration) for 24 h before harvesting. Then, each well was incubated with cell counting kit-8 (CCK-8, AR1160, Boster, China) reagent for 2 h according to the manufacturer’s instruction. Cell viability was estimated by Infinite M200PRO (Tecan, Swiss) at 450 nm.

### Apoptosis analysis

The apoptotic cells were detected by flow cytometry using annexin V-FITC/propidium iodide (PI) staining analysis kit (KGA107, Keygen, Biotech, China) as per the manufacturer’s instructions. After treatment with cisplatin or GW4869, the cells were harvested, washed, and resuspended at a final concentration of 1 × 10^6^ cells/mL. All samples were double-stained with Annexin V and PI as per the manufacturer’s instructions and analyzed by a flow cytometer (Cytomics FC 500, Beckman) equipped with CXP 2.1 software.

### Tumor xenograft assay and serum exosome isolation

In total 6–8-week-old female BALB/c nude mice (Vital River Laboratories, China) were randomly divided into two groups and maintained in pathogen-free conditions having *ad libitum* supply of filtered pathogen-free air, food, and water. SKOV3 cells, stably transfected with LV-OGT-RNAi or LV-Control-RNAi, were digested into a single-cell suspension at 1 × 10^8^/ml. In all, 100 µl of the cell suspension was subcutaneously injected into the flank of nude mice under aseptic conditions. To obtain the tumor growth curve, the tumor width and length were measured every 2 days. Tumor volume was calculated by the following formula: tumor volume = width × length^2^ × 0.5. On the 15^th^ day, mice were sacrificed, tumors were stripped, and weighed. Also, the exosomes were isolated from the mice serum. Briefly, cell debris and nonexosome organelles were removed by centrifugation at 300 × g for 10 min at 4 °C. The supernatant fractions were further centrifuged at 2000 × g for 30 min at 4 °C. Again, a second centrifugation was performed at 12,000 × g for 45 min at 4 °C. Last, the supernatants were sterile-filtered (0.22-μm filter) and from the filtrates, the exosomes were pelleted by centrifugation at 120,000 × g for 60 min at 4 °C (SW 60Ti, Beckman). All study protocols were approved by the Animal Care and Use Committee of The Forth Military Medical University.

### Statistical analysis

All data are presented as the mean ± standard error (SD) from three independent experiments, analyzed by SPSS version 17.0 software. To test the significance, a Student’s *t* test was carried out, and the *P* value was denoted by an asterisk. ^*^P < 0.05, ^**^P < 0.01 denote statistical significance of the data.

## Supplementary information

Supplementary Figure Legends

Supplementary Figure-1

Supplementary Figure-2

Supplementary Figure-3

Supplementary Figure-4

Supplementary Figure-5

Supplementary Figure-6

Supplementary Figure-7
