# Supplementary material for: Reduced O-GlcNAcylation of SNAP-23 promotes cisplatin resistance by inducing exosome secretion in ovarian cancer
Source: Cell Death Discov. 2021 May 18;7:112. doi: 10.1038/s41420-021-00489-x (PMC8128872; doi:10.1038/s41420-021-00489-x)
Supplement: Supplementary file 1 — Supplementary Figure Legends [file 41420_2021_489_MOESM1_ESM.docx]

**Supplementary figure legends**

1. A2780 and SKOV3 were transfected with control or OGT shRNA to establish stable OGT-deficient cell lines. Western blotting was used to test the expression of OGT and O-GlcNAclation in control and OGT-deficient cells. The values are presented as mean ± SD (n=3), which were three separate experiments performed in triplicate. **P < 0.01 (Student’s t test).
2. Figure 1D original uncut blots
3. Figure 2D original uncut blots
4. Figure 5B original uncut blots
5. Figure 6A, C original uncut blots
6. Figure 7A, B, C original uncut blots
7. Figure 7A, B, C original uncut blots
