# Supplementary figures and images for: Reduced O-GlcNAcylation of SNAP-23 promotes cisplatin resistance by inducing exosome secretion in ovarian cancer
Source: Cell Death Discov. 2021 May 18;7:112. doi: 10.1038/s41420-021-00489-x (PMC8128872; doi:10.1038/s41420-021-00489-x)

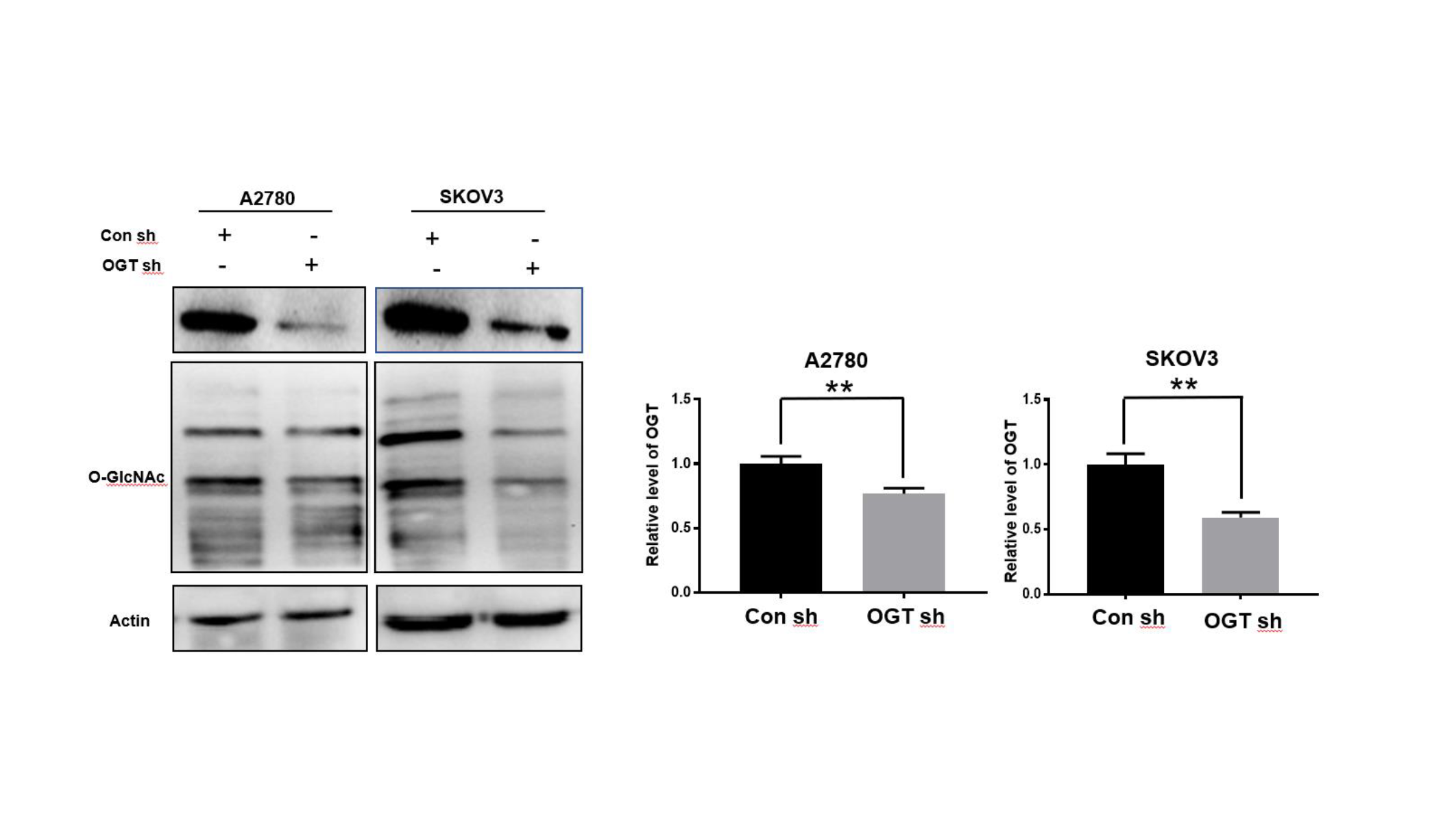

Supplement: Supplementary file 2 — Supplementary Figure-1 [file 41420_2021_489_MOESM2_ESM.png]

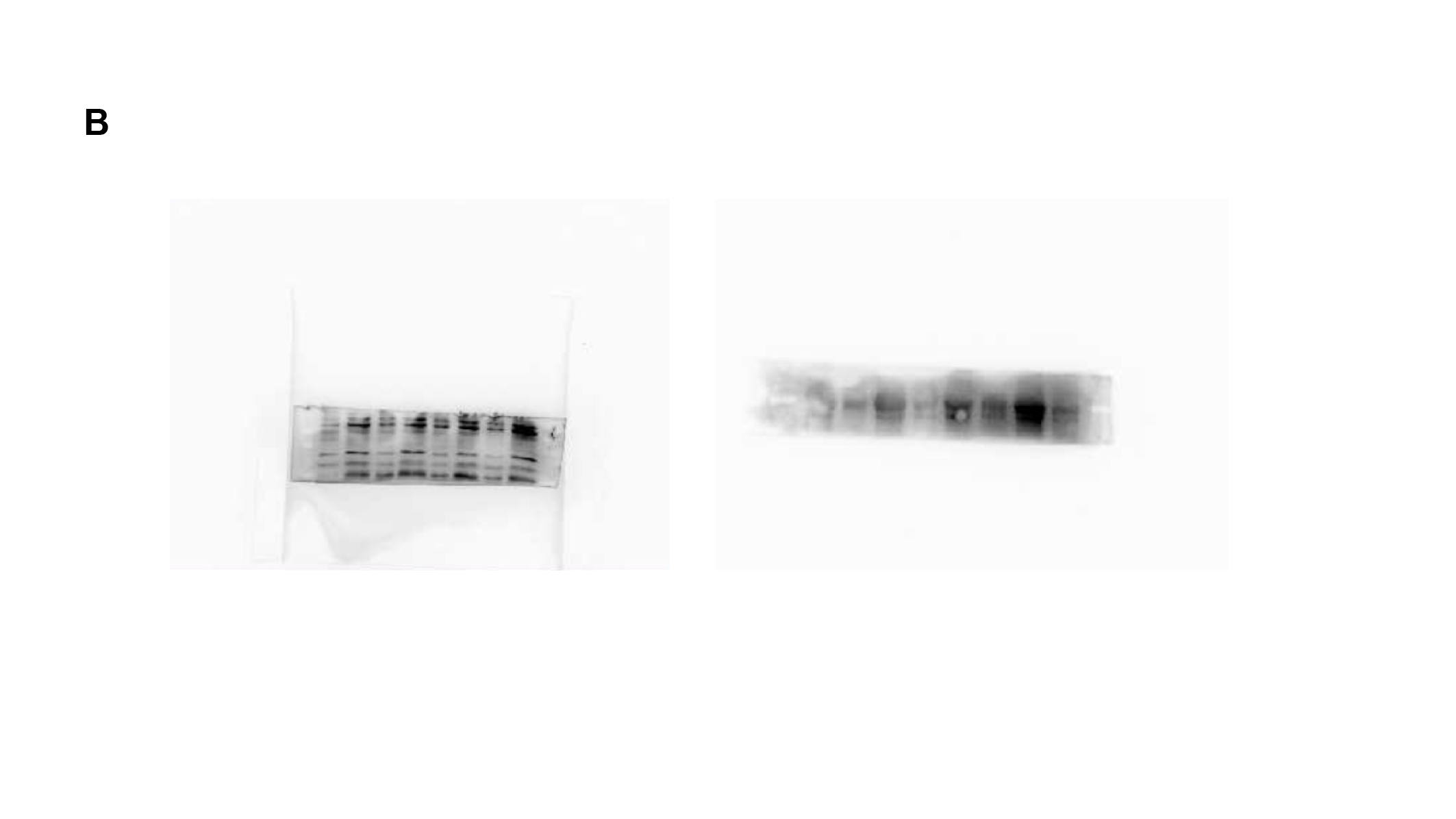

Supplement: Supplementary file 3 — Supplementary Figure-2 [file 41420_2021_489_MOESM3_ESM.png]

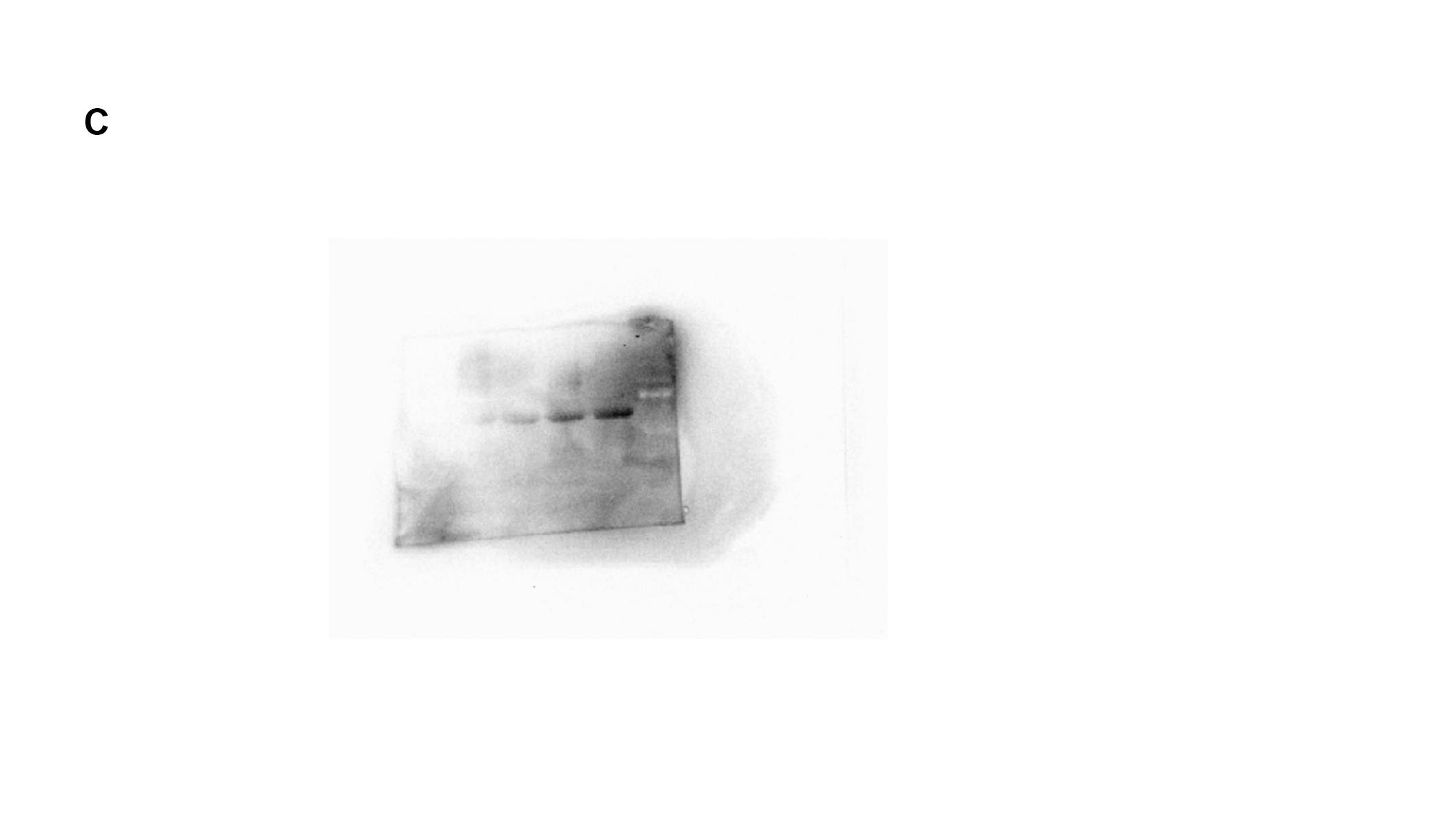

Supplement: Supplementary file 4 — Supplementary Figure-3 [file 41420_2021_489_MOESM4_ESM.png]

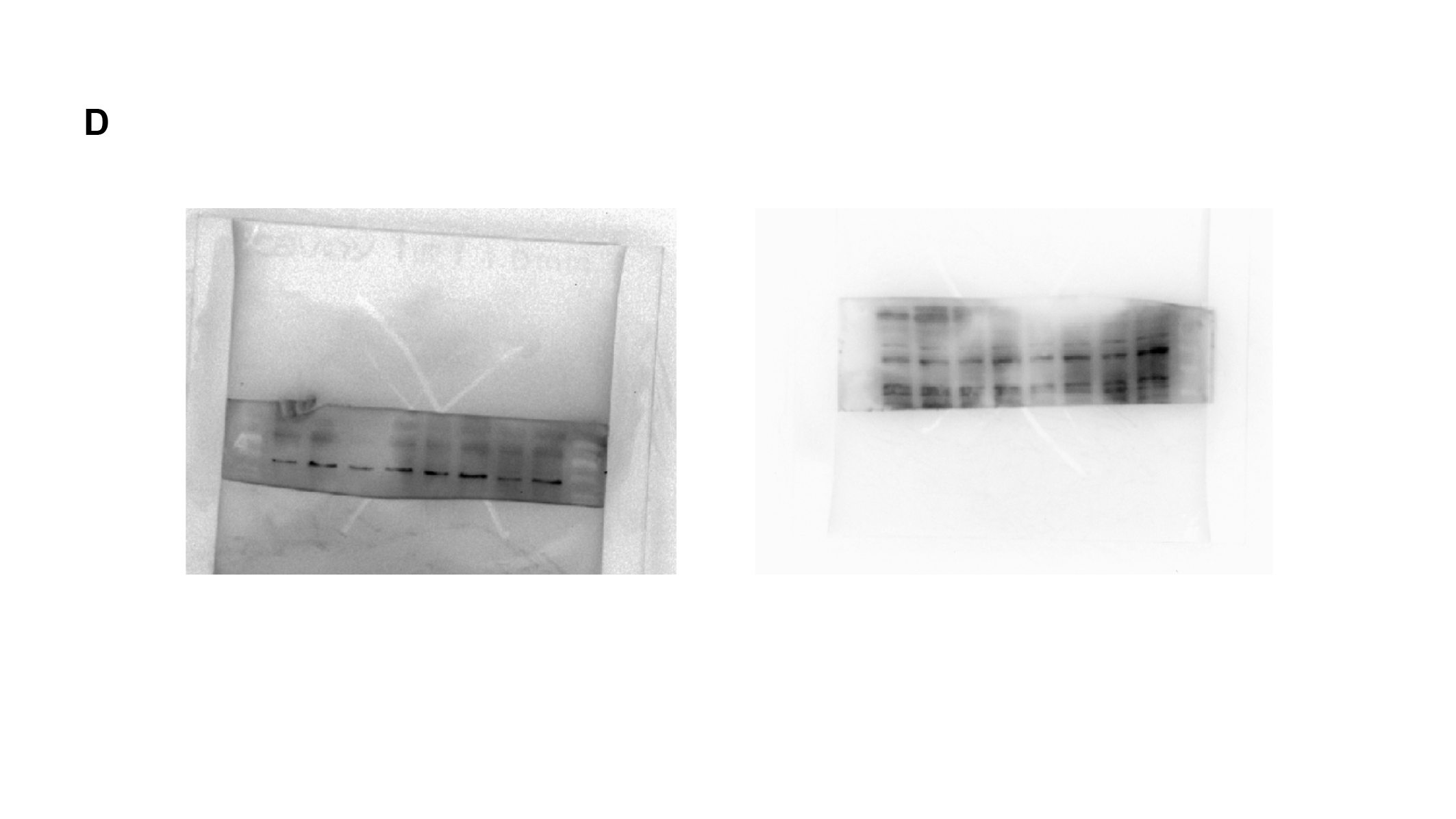

Supplement: Supplementary file 5 — Supplementary Figure-4 [file 41420_2021_489_MOESM5_ESM.png]

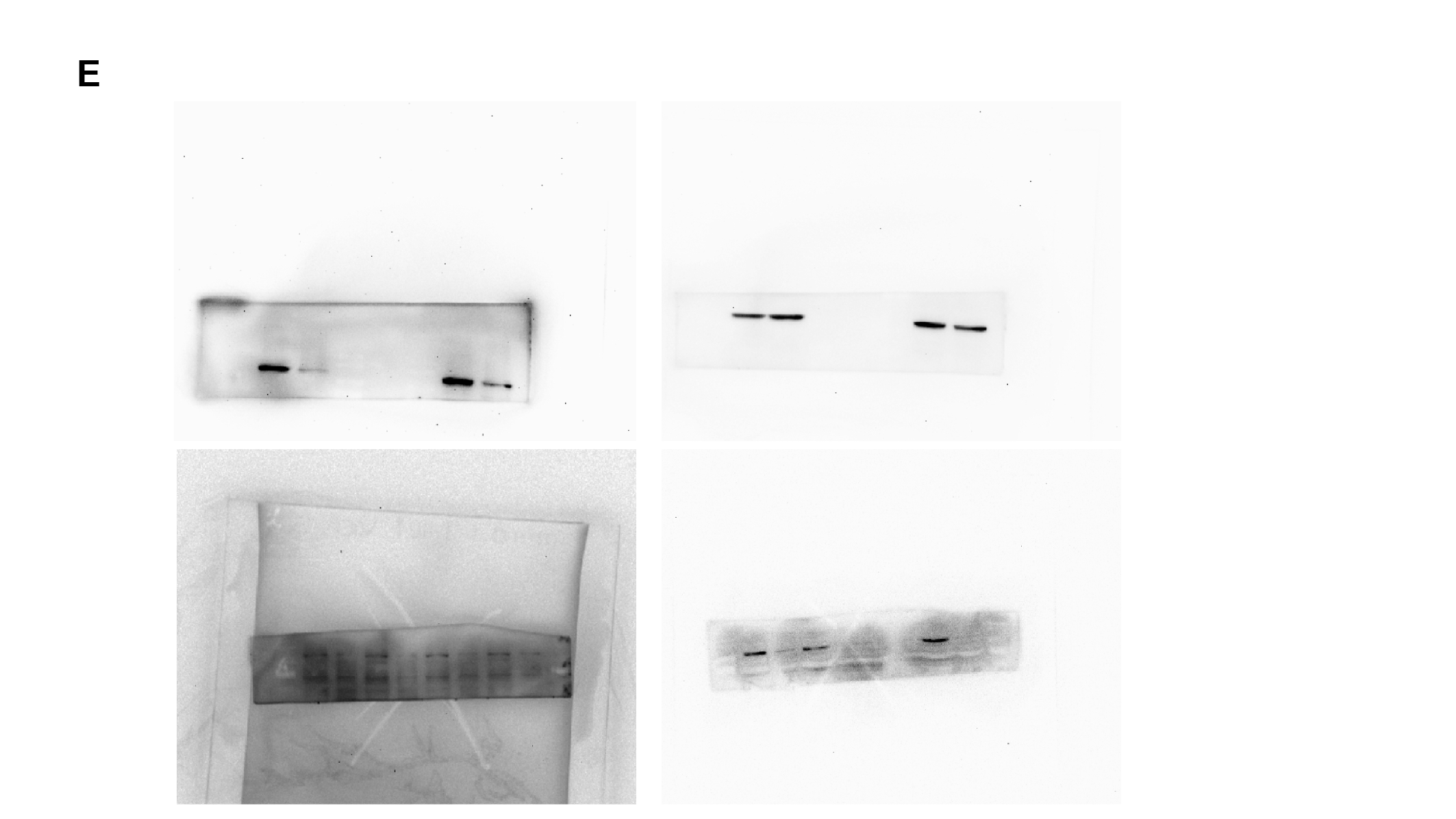

Supplement: Supplementary file 6 — Supplementary Figure-5 [file 41420_2021_489_MOESM6_ESM.png]

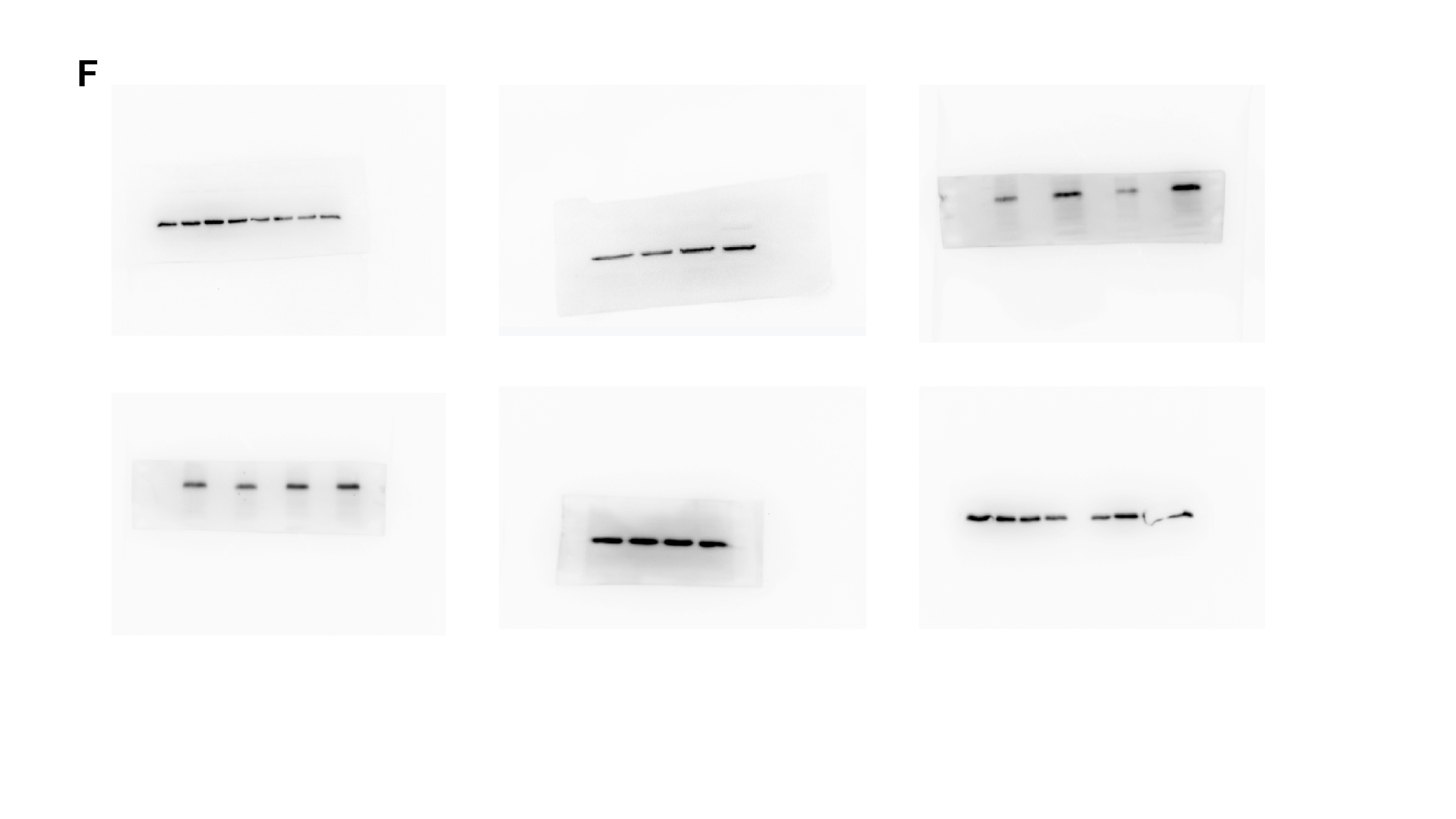

Supplement: Supplementary file 7 — Supplementary Figure-6 [file 41420_2021_489_MOESM7_ESM.png]

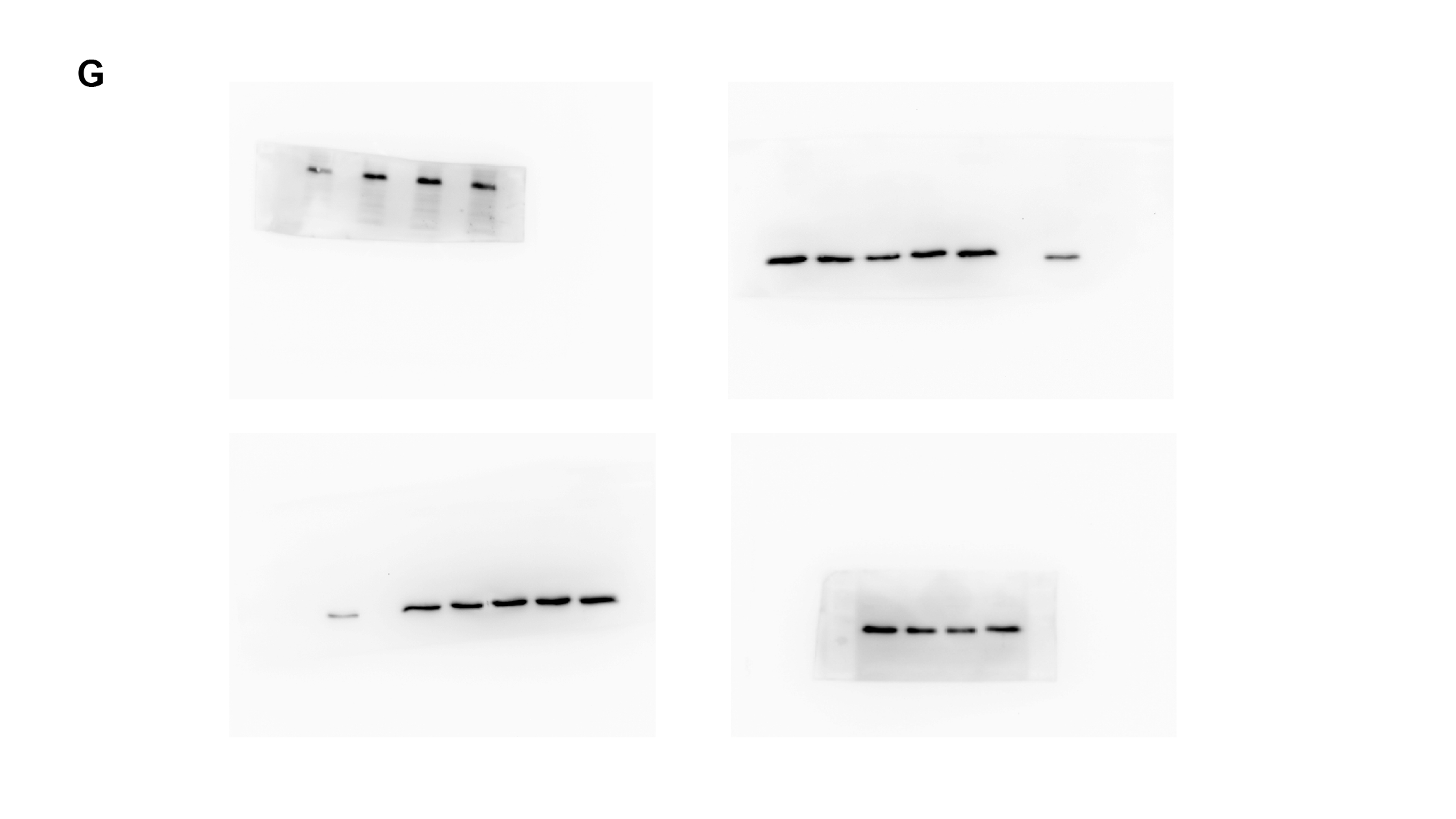

Supplement: Supplementary file 8 — Supplementary Figure-7 [file 41420_2021_489_MOESM8_ESM.png]
